# Detection Anomaly in Video Based on Deep Support Vector Data Description

**DOI:** 10.1155/2022/5362093

**Published:** 2022-05-04

**Authors:** Bokun Wang, Caiqian Yang, Yaojing Chen

**Affiliations:** ^1^College of Civil Engineering and Mechanics, Xiangtan University, Xiangtan 411100, China; ^2^School of Civil Engineering, Southeast University, Nanjing 210096, China; ^3^Jiangsu Expressway Engineering Maintenance Co., Ltd., Huaian 223005, China

## Abstract

Video surveillance systems have been widely deployed in public places such as shopping malls, hospitals, banks, and streets to improve the safety of public life and assets. In most cases, how to detect video abnormal events in a timely and accurate manner is the main goal of social public safety risk prevention and control. Due to the ambiguity of anomaly definition, the scarcity of anomalous data, as well as the complex environmental background and human behavior, video anomaly detection is a major problem in the field of computer vision. Existing anomaly detection methods based on deep learning often use trained networks to extract features. These methods are based on existing network structures, instead of designing networks for the goal of anomaly detection. This paper proposed a method based on Deep Support Vector Data Description (DSVDD). By learning a deep neural network, the input normal sample space can be mapped to the smallest hypersphere. Through DSVDD, not only can the smallest size data hypersphere be found to establish SVDD but also useful data feature representations and normal models can be learned. In the test, the samples mapped inside the hypersphere are judged as normal, while the samples mapped outside the hypersphere are judged as abnormal. The proposed method achieves 86.84% and 73.2% frame-level AUC on the CUHK Avenue and ShanghaiTech Campus datasets, respectively. By comparison, the detection results achieved by the proposed method are better than those achieved by the existing state-of-the-art methods.

## 1. Introduction

In order to improve the safety of public life and assets, video surveillance systems have been widely deployed in public places such as shopping malls, hospitals, banks, and streets. In most cases, how to detect video abnormal events in a timely and accurate manner is the main goal of social public safety risk prevention and control. Video abnormal events are defined as abnormal or irregular patterns in the video that do not conform to normal patterns. These incidents often include fights, riots, violations of traffic rules, trampling, holding arms, and abandoning luggage. However, due to the ambiguity of anomaly definitions, the scarcity of anomalous data, and the complex environmental background and human behavior, video anomaly detection is a major problem in the field of computer vision. In a nutshell, most of the current research work on video anomaly detection can be divided into two steps, such as feature extraction and normal model training [1]. Feature extraction can be achieved by manual technology or automatic feature extraction technology (representation learning or features based on deep learning). In normal model training, normal samples are used for learning, and then samples that do not conform to the learned model are judged as abnormal events. Then, the classification according to features can be divided into three different methods [2]. The first type is the trajectory-based methods [3]. This type of method obtains trajectory features by tracking the target. However, in dense scenes, the target tracking is a big problem. The second type of methods is based on global features [4, 5]. This type of method takes the video frame as a whole and extracts some low-level or middle-level features such as spatiotemporal gradients and optical flow. In a moderately crowded and dense environment, these methods can keep effective. The third type is the grid feature-based methods [6]. This type of method often divides the video frame into multiple small grids through dense sampling and then extracts the underlying features of a single grid because each grid can be individually evaluated. According to different normal model training methods, the present methods can also be divided into three different types. The first type is the cluster-based method [7]. This type of method is often based on an assumption that the normal sample belongs to a category or is relatively far from the cluster center. The abnormal samples do not belong to any category or are far away from the cluster center and then cluster the normal samples to build the model. The second is the method based on sparse reconstruction [8, 9]. This type of method assumes that the sparse linear combination of patterns can represent normal activities with the smallest reconstruction error. Because there is no abnormal activity in the training data set, it can represent abnormal patterns with a large reconstruction error. The third type is probabilistic model-based methods. This method considers that normal samples conform to a certain probability distribution, while abnormal samples do not conform to this distribution.

Recently, the latest progress of deep learning has proved the obvious advantages of deep learning-based methods in many computer vision applications [10]. As one of the tasks in computer vision, video anomaly detection is no exception. Different from traditional manual feature-based methods, deep learning methods often use pretrained networks to extract high-level features from videos or use existing network structures to establish end-to-end anomaly detection models based on normal models. For the former idea [11, 12], there is not much difference between the two steps of traditional abnormal event detection. For the latter idea [13–16], the two steps of feature extraction and model building are often jointly optimized in a deep network. These end-to-end deep networks include Auto-Encoder (AE), Deep Siamese Network (DSN), and Generative Adversarial Nets (GANs) [14, 17–20]. However, these network models are often designed for other tasks such as generative models and compression, rather than for anomaly detection tasks.

In the framework of deep learning, this paper proposes a new anomaly detection method based on Deep Support Vector Data Description (DSVDD) for anomaly detection tasks. Through DSVDD, not only can the smallest size data hypersphere be found to establish SVDD but also useful data feature representations and normal models can be learned. To this end, DSVDD uses a jointly trained deep neural network to map normal sample data to the smallest volume hypersphere. Then, in the test, the samples mapped inside the hypersphere are judged as normal, while the samples mapped outside the hypersphere are judged as abnormal. The RGB graph and the optical flow graph are composed of a 6-channel data and directly input into a DSVDD model; that is, it can detect the appearance abnormality and movement abnormality at the same time. The experimental results on the two public data sets of Avenue [9] and ShanghaiTec [17] show that the detection results of the method proposed in this paper are excellent, which exceed the state of the art.

## 2. Principle of Algorithm

**Figure 1:**
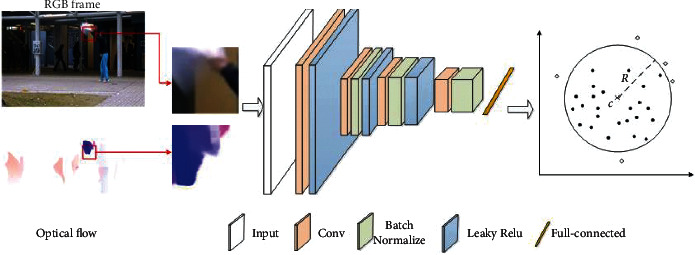
The flow chart of video anomaly detection based on DSVDD.

The overall process of the method proposed in this paper is shown in Figure 1. In the training phase, the RGB images and optical flow diagrams of the training samples are intensively sampled and then merged into a 6-channel data to train the DSVDD model. In the testing phase, the RGB image and optical flow diagram composition of the video frame to be tested are also obtained after inputting the 6-channel data into the learned DSVDD model. It is determined whether the area is abnormal. In this section, the principle of SVDD is first briefly introduced, and then the training and testing process of video abnormal events based on DSVDDD is described.

### 2.1. SVDD

SVDD is a description method based on boundary data (support vector). Its goal is to find a hypersphere that contains all or almost all training samples and has the smallest volume (the center is *c* ∈ ℱ_*k*_, and the radius is *R* > 0). In fact, the SVDD optimization problem can be transformed as follows:

(1) $$\begin{array}{c} \underset{R,W}{\min}\;R^{2}+\frac{1}{vn}\sum_{i}\xi_{i}\\ \text{s.t}.\;{\left\|\phi_{k}\left(x_{i}\right)-c\right\|}_{{ℱ}_{k}}^{2}\leq R^{2}+\xi_{i},\;\xi_{i}\geq 0. \end{array}$$

In (1), the slack variable allows a soft boundary; *ξ*_*i*_ ≥ 0 and *v* ∈ (0,1] are hyperparameters to control the balance between the penalty term and the volume edge of the hypersphere. Therefore, a point that falls outside the hypersphere, such as ‖*ϕ*_*k*_(*x*_*i*_) − *c*‖_ℱ_*k*__^2^ > *R*^2^, is decided to be abnormal. SVDD has been widely used in fields such as anomaly detection, face recognition, speech recognition, image restoration, and medical imaging [21].

### 2.2. DSVDD

DSVDD learns a deep neural network *ϕ*(*·*; *𝒲*) with the weight *𝒲*, so that the input normal sample space can be mapped to a hypersphere with the center and radius of the smallest. The normal sample is mapped in the hypersphere *𝒳*⊆*ℝ*^*d*^, and the abnormal sample is mapped on the hypersphere.

Specifically, for the sample area input space *𝒳*⊆*ℝ*^*d*^ and output space ℱ⊆*ℝ*^*p*^, a neural network with *L* ∈ *ℕ* hidden layers can project the input space to the output space *𝒳*⟶ℱ, where *𝒲*={*W*^1^, *W*^2^,…, *W*^*L*^} are the weights of the hidden layers *ℓ*={1,2,…, *L*} correspondingly. Therefore, *ϕ*(*x*; *𝒲*) ∈ ℱ is the characteristic representation of the input sample *x* ∈ *𝒳*. The goal of the DSVDD method is to jointly optimize the network weights *𝒲* and the output space to meet the minimum hyperspherical constraints of the center *c* and the radius *R*. Then, given the training sample *𝒟*_*n*_={*x*_1_, *x*_2_,…, *x*_*n*_}, the soft-boundary objective function of DSVDD is as follows:

(2) $$\begin{array}{c} \underset{R,W}{\min}\;R^{2}+\frac{1}{vn}\sum_{i=1}^{n}\max\left\{0,{\left\|\phi \left(x_{i};W\right)-c\right\|}^{2}-R^{2}\right\}\\ +\frac{\lambda }{2}\sum_{\ell =1}^{L}{\left\|W\right\|}_{F}^{2}. \end{array}$$

For (2), in the SVDD method, the minimization of *R*^2^ means to minimize the volume of the hypersphere. The second item is the penalty items that are mapped out of the hypersphere through the neural network, such as those from the center of the hypersphere ‖*ϕ*(*x*_*i*_; *𝒲*) − *c*‖ greater than radius *R*. The hyperparameter *v* ∈ (0,1] controls the balance between the volume of the hypersphere and the deviation of the boundary, which allows certain points to be mapped to the outside of the sphere. The last item is the network parameter weight *𝒲* the attenuation regularization term, where *λ* > 0 and ‖·‖_*F*_ represents the Frobenius norm.

The optimization of (2) enables the network to learn weights *𝒲*, so that the data points can be closely projected to the center of the hypersphere **c** nearby. For this reason, the deep network must extract the common factors of data changes. In fact, normal samples can often be mapped closer to the center of the hypersphere *c*, while abnormal samples are mapped farther from the center or outside the hypersphere. In this way, a compact description of the normal model is obtained.

In actual tasks, it is often assumed that the training samples are all normal samples, so the objective function can be simplified to a single-class classification problem as follows:

(3) $$\begin{array}{c} \underset{W}{\min}\;\frac{1}{n}\sum_{i=1}^{n}{\left\|\phi \left(x_{i};W\right)-c\right\|}^{2}+\frac{\lambda }{2}\sum_{\ell =1}^{L}{\left\|W\right\|}_{F}^{2}. \end{array}$$

DSVDD simply uses a secondary loss to punish the distance of each deep network representation *ϕ*(*x*_*i*_; *𝒲*) and **c**. The second term is the regularization term of network parameter weight attenuation *𝒲*, *λ* > 0. Equation (3) can also be regarded as a hypersphere with the smallest volume as the center. However, unlike Equation (2) using a soft boundary, Equation (3) shrinks the sphere by minimizing the average distance from the center of all data representations, instead of directly penalizing the radius and data representation that falls outside the sphere. Similarly, in order to map the samples as close to the center of the hypersphere as possible, the deep neural network must extract the changing common factors.

The weights *𝒲* of the neural network in DSVDD can be optimized by common back propagation methods (such as stochastic gradient descent). Because the network weight *𝒲* and hypersphere radius *R* are with different scales, it is impossible to optimize DSVDD with one learning rate. Therefore, it is necessary to alternately optimize the network weights *𝒲* and hypersphere radius *R* by the alternate minimization/block coordinate descent method.

### 2.3. Test Phase

Given test sample area *x*′ ∈ *𝒳*, the anomaly score can *e* calculated as follows:

(4) $$\begin{array}{c} s\left(x^{\prime }\right)={\left\|\phi \left(x^{\prime };W^{*}\right)-c\right\|}^{2}. \end{array}$$

where *𝒲*^*∗*^ are the trained network model parameters. It is worth noting that network parameters can fully describe the DSVDD model. And predictions can be made without storing any data, so DSVDD has a very low storage complexity. Therefore, the computational complexity during testing is small.

In order to infer whether the test sample area is an abnormal sample, thresholds can be set on *s*(*x*′) to make judgments as follows:

(5) $$\begin{array}{c} s\left(x^{\prime }\right)\overset{\text{abnormal}}{\underset{\text{normal}}{≷}}\theta , \end{array}$$

where *θ* is the threshold that determines the sensitivity of the detection method in this paper.

## 3. Experiment

### 3.1. Dataset

This paper evaluates the performance of the DSVDD method on two publicly available data sets, i.e., the Avenue data set [9] and ShanghaiTech data set [17]. The Avenue data set is one of the most widely used benchmarks for video anomaly detection. It contains 16 training video clips and 21 test video clips, including 47 abnormal incidents that occurred on the streets of the Chinese University of Hong Kong. Each video is about 1 minute long and has a resolution of 640 × 360. Normal events are walking on the street, and abnormal events include running, loitering, and throwing. ShanghaiTech data set [17] is one of the largest newly proposed datasets for video anomaly detection. Unlike other data sets, the video clips in this data set come from 13 different cameras with different lighting conditions and camera angles. It has 330 training video clips and 107 test video clips containing 130 abnormal events. The resolution of the video frame is 856 × 480. Abnormal events in this data set include chasing and noise.

### 3.2. Evaluation Index

According to previous work [14], this paper calculates the frame-level receiver operating characteristic (ROC) curve and uses the area under the curve (AUC) score as an evaluation indicator. A higher AUC score indicates better anomaly detection performance. If an area in the video frame is judged to be abnormal, the frame is judged to be abnormal. We first obtain the anomaly scores of all video frames and then calculate the frame-level AUC scores.

### 3.3. Supplementary Details

For the two data sets, each frame is adjusted to a size of 320 × 240, and the optical flow image is calculated by the RAFT optical flow method provided in [22] through a network pretrained on the things data set. The original video frame and the calculated optical flow graph are combined into a 6-channel data, then cropped into 16 × 12 grid images according to the size of 20 × 20, and then input into DSVDD for training and prediction. The deep neural network part of DSVDD is in accordance with Conv (16, 3 × 3)-Leaky ReLU-ConvTran (32, 3 × 3)-BN-Leaky ReLU-ConvTran (64, 3 × 3)-BN-Leaky ReLU–FullyConnectd64 structure. In the training phase, the batch size is set to 128, the initial learning rate is 0.0003, the weight decay is 0.0001, and the training is performed 1000 iterations.

### 3.4. Experimental Results

This section compares the proposed DSVDD with the results obtained by several latest methods that only use positive sample training. These methods include Conv-AE [14], Stacked RNN [17], Unmasking [18], Davide et al. [19], Object-centric auto-encoder [20], MemAE [23], and New Baseline [24].  Table 1 lists the evaluation results of these methods on the frame-level anomaly detection on the two data sets [25–27].

On the Avenue dataset, the DSVDD method proposed in this paper is superior to the results obtained by other methods, with an AUC score of 87.4%, which is 2.3% higher than the baseline method proposed in 2018 [24]. As far as we know, in terms of the frame-level AUC scores of all test videos in this data set, the DSVDD proposed in this paper has achieved the best results. It is worth noting that the Object-centric auto-encoder [20] method achieved 89.3% of the frame-level AUC in their paper, but this is calculated through different indicators in their paper and the actual calculation of Object-centric. The frame-level AUC score obtained by the auto-encoder [20] method should be 86.5%, which is 0.9% lower than the method proposed in this paper.

On the ShanghaiTech dataset, the method DSVDD proposed in this paper achieves a frame-level AUC score of 74.5%, which is 1.7% higher than the baseline method proposed in 2018 [24] and second only to Object-centric auto-encoder [20]. The method achieved 78.5%. The Object-centric auto-encoder [20] method uses an object detection-based method for anomaly detection, and its performance largely depends on the output of its object detection algorithm. Therefore, detection-based methods cannot determine abnormal events that have not occurred before, and this often occurs in abnormal detection. Similarly, the MemAE method [23] requires the help of a pretrained pose estimator to achieve better results, so it is limited to detecting abnormal events related to people. In contrast, the DSVDD method proposed in this article does not have this limitation and is very reliable when applied to various scenarios. Obviously, in addition to these two specially limited methods, the DSVDD method proposed in this paper is at least 1.7% ahead of other methods in frame-level AUC.

**Figure 2:**
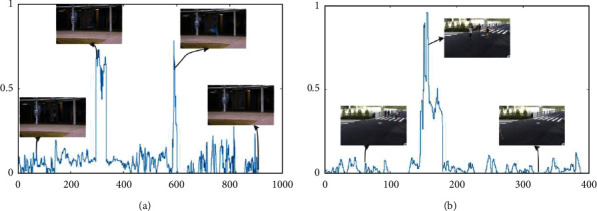
Illustration of detection results on different datasets. (a) Example of detection result on the Avenue data set (b) Example of detection result on the Avenue data set.

In Figure 2, some examples of abnormal score curves in the method proposed in this paper are shown, and some key frames with normal or abnormal events are given. Among them, the abscissa is the number of video frames, and the ordinate anomaly score has been normalized to 1. It can be seen that in the two data sets, the method proposed in this paper can correctly distinguish between normal and abnormal events. If an abnormal event occurs suddenly, such as running as shown in Figure 2(a), the abnormal score will increase sharply. If the abnormal event occurs slowly, as shown in Figure 2(b), the abnormal score will gradually increase. If the object that caused the abnormality disappears from the camera's field of view, the abnormality score will quickly decrease to close to 0.

## 4. Conclusion

In this paper, a video anomaly detection method based on DSVDD is proposed. DSVDD can be seen as a combination of deep learning and SVDD. It uses a jointly trained deep neural network to map normal sample data to the smallest volume hypersphere. Then, in the test, the samples mapped inside the hypersphere are judged as normal, while the samples mapped outside the hypersphere are judged as abnormal. A large number of experimental results on two public data sets show that the proposed method is significantly better than the existing methods, which proves the effectiveness of the anomaly detection method proposed in this paper. In the future, we will reduce the computational complexity on the basis of ensuring the accuracy of the algorithm and focus on improving the real-time performance of the algorithm to better apply it to actual scenarios. [25–27].

## Figures and Tables

**Table 1:** AUC scores of the anomaly detection results.

Method	Avenue	ShanghaiTech campus
Conv-AE	80.0%	60.9%
Stacked RNN	81.7%	68.0%
Unmasking	80.6%	—
Davide et al.	—	72.8%
Object-centric auto-encoders	86.5%	78.5%
MemAE	83.3%	72.2%
New baseline	85.1%	72.8%
Ours	86.8%	73.2%

## Data Availability

The datasets used in this paper can be accessed upon request.
